# Effect of Brazil Nuts on Selenium Status, Blood Lipids, and Biomarkers of Oxidative Stress and Inflammation: A Systematic Review and Meta-Analysis of Randomized Clinical Trials

**DOI:** 10.3390/antiox11020403

**Published:** 2022-02-16

**Authors:** Justyna Godos, Francesca Giampieri, Agnieszka Micek, Maurizio Battino, Tamara Y. Forbes-Hernández, José L. Quiles, Nadia Paladino, Luca Falzone, Giuseppe Grosso

**Affiliations:** 1 Department of Biomedical and Biotechnological Sciences, University of Catania, 95123 Catania, Italy; justyna.godos@gmail.com (J.G.); nadia.paladino97@gmail.com (N.P.); 2 Research Group on Food, Nutritional Biochemistry and Health, Universidad Europea del Atlántico, 39011 Santander, Spain; f.giampieri@univpm.it (F.G.); jlquiles@ugr.es (J.L.Q.); 3 Institute of Nursing and Midwifery, Faculty of Health Sciences, Medical College, Jagiellonian University, 31-501 Krakow, Poland; agnieszka.micek@uj.edu.pl; 4 Department of Clinical Sciences, Polytechnic University of Marche, 60131 Ancona, Italy; m.a.battino@univpm.it; 5 International Joint Research Laboratory of Intelligent Agriculture and Agri-Products Processing, Jiangsu University, Zhenjiang 212013, China; 6 Biomedical Research Centre, Department of Physiology, Institute of Nutrition and Food Technology “José Mataix”, University of Granada, 1800 Granada, Spain; tforbes@uvigo.es; 7 Epidemiology Unit, IRCCS Istituto Nazionale Tumori “Fondazione G. Pascale”, 80131 Naples, Italy; l.falzone@istitutotumori.na.it

**Keywords:** Brazil nuts, selenium, glutathione peroxidase, antioxidant, oxidative stress, cholesterol, blood lipids, meta-analysis, clinical trial

## Abstract

Tree nuts, including Brazil nuts, have been hypothesized to impact cardiovascular health through the modulation of oxidative stress and inflammation. Nonetheless, a quantitative analysis of these effects has not been performed. Therefore, the aim of this study was to systematically revise and quantify the effect of Brazil nut intervention on selenium status, blood lipids, and biomarkers of oxidative stress and inflammation using a meta-analytical approach. To meet the goals of this study, a systematic search of PubMed, EMBASE, and Web of Science databases of published randomised clinical trials reporting on dietary interventions with Brazil nuts and their effects on selenium status, blood lipids, and markers of oxidative stress and inflammation was performed. Eight articles were included for systematic review and meta-analysis. Based on the conducted analysis, a significant positive effect of Brazil nuts on selenium blood concentration (SMD = 6.93, 95% CI: 3.99; 9.87) was found. Additionally, a positive effect of Brazil nut intervention on glutathione peroxidase activity (SMD = 0.53, 95% CI: 0.07; 0.99) was observed. However, no significant results were found when considering blood lipid levels, including results for total cholesterol (SMD = −0.22, 95% CI: −0.57; 0.14), HDL cholesterol (SMD = −0.04, 95% CI: −0.28; 0.19) and LDL cholesterol (SMD = −0.15; 95% CI: −0.43; 0.13). In conclusion, the findings from this study suggest that Brazil nut consumption improves selenium status and exerts antioxidant effects, which could be considered a potential pathway for the prevention of metabolic disorders related to altered blood lipid profiles. However, further studies are needed to elucidate the effect of Brazil nuts toward blood lipid profile, also preferably controlling for other biomarkers.

## 1. Introduction

Metabolic impairment is characterized by an imbalance of homeostasis of the human body in favour of increased visceral fat, dyslipidaemia, compromised blood pressure and glucose/insulin regulation, and low-grade chronic inflammation [1]. The oxidative stress state exacerbated in these conditions is secondary to the release of cytokines, such as tumour necrosis factor-alpha (TNF-alpha) and interleukin-6 (IL-6), leading to increased production of reactive oxygen species (ROS), which have been considered a possible pathogenic mechanism underlying cardiovascular comorbidities [2]. Moreover, oxidative stress increases the oxidation of low-density lipoprotein (LDL) particles, which in turn causes injury of the endothelium, contributing to an increased risk of atherosclerosis [3].

The human body is equipped with an antioxidant system to counteract such processes, but environmental factors, such as nutrient intake, seem to play a key role in enhancing the natural defences of the organism [4,5]. Selenium is an essential micronutrient that functions as a component of many selenoproteins in antioxidant and redox reactions. In particular, selenium is necessary for the functioning of glutathione peroxidase (GPx), which is an antioxidant enzyme that catalyses hydrogen peroxide and lipid hydroperoxide through glutathione metabolism [6]. While GPx protects membrane lipids and other cellular and extracellular components from oxidative damage, selenium has been hypothesized to exert anti-atherogenic effects by lowering oxidative stress in the endothelium.

The major dietary sources of selenium include Brazil nuts (0.2–512 μg/g), yeast (500–4000 μg/g) and fish (0.06–0.63 μg/g) [7]. Brazil nuts (*Bertholletia excelsa*, family *Lecythidaceae*) are South American tree nuts characterized by a high content in several bioactive substances, such as phenolic compounds, tocopherol, folate, magnesium, potassium, calcium, proteins and mono- (MUFA) and polyunsaturated (PUFA) fatty acids, but are best known as one of the major food sources of selenium [8]. It has been demonstrated that consumption of tree nuts lowers total cholesterol, LDL cholesterol, ApoB, and triglycerides [9], affects energy metabolism [10], modulates vascular tone [11], and lowers the risk of cardiovascular outcomes [12]. However, data to corroborate the potential beneficial effects of Brazil nuts on selenium status and their antioxidant effect are lacking. The aim of the present study was to assess from existing literature whether Brazil nuts significantly improve selenium status and have an effect on markers of oxidative stress and inflammation, as well as lipid profile.

## 2. Materials and Methods

The design, analysis, and reporting of this study followed Preferred Reporting Items for Systematic Reviews and Meta-Analyses (PRISMA) (Supplementary Table S1) [13]. Furthermore, eligibility criteria for the systematic review and meta-analysis were specified using the PICOS approach: determination of the population (P), intervention (I), comparison (C), outcomes (O), and study design (S) (Table 1).

### 2.1. Systematic Search and Study Selection

A systematic search for all studies examining Brazil nut intervention and selenium blood levels, lipid profile as well as markers of inflammation and oxidative stress was performed using PubMed, EMBASE, and The Cochrane Library from their inception to April 2021, with restriction to titles and abstracts. Search terms included: (nuts OR nut OR Brazil nuts OR Brazil nut) AND (selenium OR glutathione peroxidase OR GPx OR glutathione OR GSH OR GSSG OR inflammation OR inflammatory OR cytokines OR cytokine OR C-reactive protein OR CRP OR interleukin-6 OR IL-6 OR tumour necrosis factor OR TNF-alpha OR antioxidant OR oxidative stress OR oxidation OR oxidative status OR antioxidant capacity OR total antioxidant capacity OR TAC OR lipid oxidation OR lipid peroxidation OR 8-epi-PGF2a OR malondialdehyde OR MDA OR ferric reducing ability of plasma OR FRAP OR ORAC OR lipid profile OR HDL OR LDL OR cholesterol OR total cholesterol OR lipid OR triglycerides OR apolipoprotein). Additional hand-searches of the reference list of all included studies and reviews were performed. Randomized controlled clinical trials with crossover or parallel design reporting on the effect of Brazil nuts on selenium blood levels, lipid profile or markers of inflammation and oxidative stress were considered eligible. Studies with the administration of Brazil nut derivatives characterized by a diverse nutrient profile (i.e., Brazil nut oil) or without clear specification of the administered doses (i.e., recommendation to increase Brazil nut intake) or with single administrations were excluded. When duplicate publications were identified, the report including most complete data for each endpoint of interest was selected for quantitative analysis. The process of systematic search and study selection was executed by two authors (A.M. and J.G.), and any discrepancies were discussed and resolved by consensus.

### 2.2. Data Extraction

Main study characteristics and data for all endpoints were extracted from the studies that met inclusion criteria. The following information was collected: first author name and publication year, study design, country, population characteristics, sample size for intervention and control group, sex and mean age of participants, type and duration of intervention, and outcomes of interest. Additionally, means and standard deviations (SDs) for baseline values, end values, and differences in change from baseline to end of intervention were recorded for all endpoints. Missing information for any endpoint data was requested from the authors. In case requested data were not provided, an attempt to calculate or estimate them according to the methods recommended by Cochrane was made.

### 2.3. Risk of Bias Assessment

The Cochrane Risk of Bias Tool was used to assess risk of bias in individual studies. Briefly, the tool allows for scoring of the studies based on: bias arising from the randomization process, bias due to deviations from intended interventions, bias due to missing outcome data, bias in the measurement of the outcome, and bias in the selection of the reported results. The final judgment categories are: low risk of bias when all the domains score as having low risk of bias; unclear risk of bias when the study is considered to raise some concerns in at least one domain but not to be at high risk of bias for any domain; and high risk of bias when the study is considered to be at high risk of bias in at least one domain or is considered to have some concerns for multiple domains in a way that considerably lowers confidence in the result [14].

### 2.4. Data Synthesis and Statistical Analysis

Analyses were conducted using the generic inverse variance method with random-effects models. The presence of between-studies heterogeneity was assessed using the Q statistic (significance set at *p* < 0.10) and was quantified by the I2 statistic with the following categorisation of heterogeneity: <50% “low”; 50–75%, “substantial”; and ≥75%, “high”.

To reflect the group differences rather than individual changes, the raw-score metric was applied taking into account variability within each intervention group. To harmonize the same outcome often measured using different methods or expressed in different units, the population effect size was estimated as the difference of standardized mean changes (SMD) between groups [15]. Plasma or serum concentrations (highly correlated with the alternative choices, e.g., measurements in erythrocytes) were extracted preferentially in the cases when many measurements of the individual outcome were available in one trial. In the studies with insufficient information to calculate correlations between measurements before and after intervention (or equivalently the SDs for changes), three possible values of missing correlations were consecutively imputed (0.3, 0.5 and 0.7) to check whether different methods of handling missing data can yield different results. The main analysis was conducted with imputed missing correlations of 0.5 and for both crossover and parallel design studies under the assumption of providing equivalent estimates of the treatment effect [16]. Several sensitivity analyses were performed to check the robustness of the findings. First, identified crossover trials were excluded due to different paired nature design, and second, the alternative values of missing correlations between repeated measurements were imputed. Performing a separate meta-analysis based exclusively on crossover trials was impossible due to the limited number of the studies; thus, we described the main findings only by a narrative approach. To determine whether individual trials exerted an undue influence on the overall results, influential analyses by excluding each study one at the time were performed. Meta-regression analysis was conducted according to daily intake of selenium contained in Brazil nuts, duration of follow-up, mean age of participants, percentage of men, sample size and year of publication. Publication bias was assessed by visual inspection of funnel plots and was formally complemented by Egger’s tests. In all analyses, a two-sided *p* value 0.05 was set as the level of significance. Data were analysed using R software version 4.0.2 (Development Core Team, Vienna, Austria).

## 3. Results

**Figure 1:**
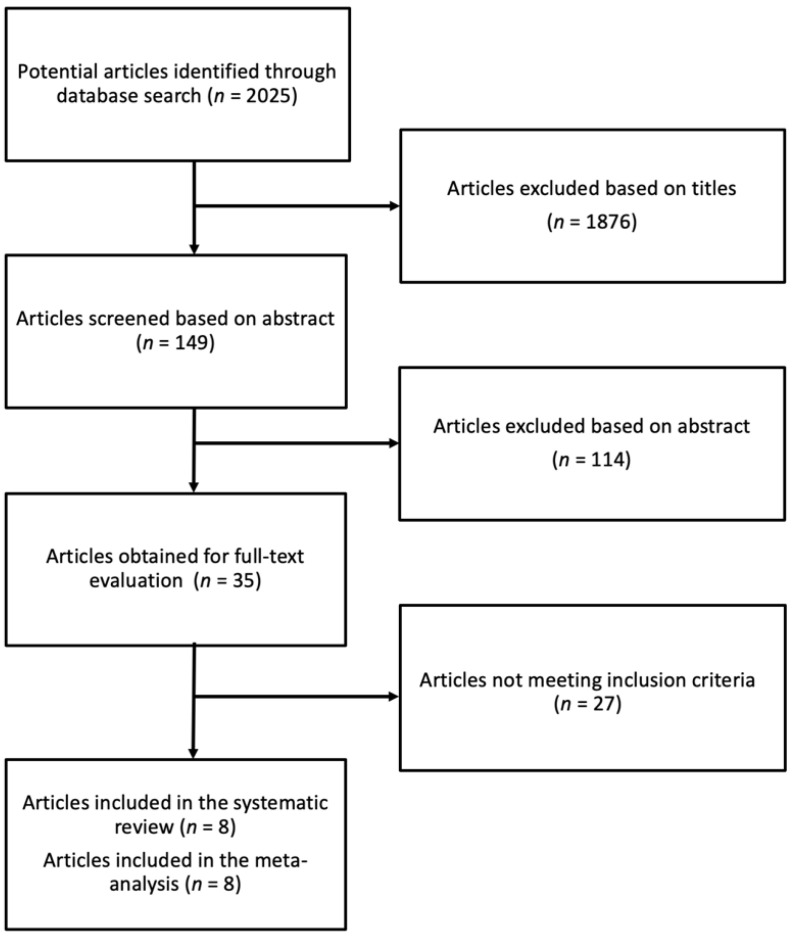
Flow chart of the study selection process.

The systematic literature search identified 2025 studies, out of which 1876 were excluded on the basis of title, with 114 after abstract revision. Of the remaining 35 publications left for full-text reading, 27 were excluded due to the following reasons: (i) unclear dietary regimens or not quantifiable Brazil nut intervention, (ii) data overlapping with another study, (iii) lack of randomization, (iv) investigating the effect of a single administration, (v) in vivo study design, and (vi) lack of placebo/control group. The article selection process is shown in Figure 1.

**Figure 2:**
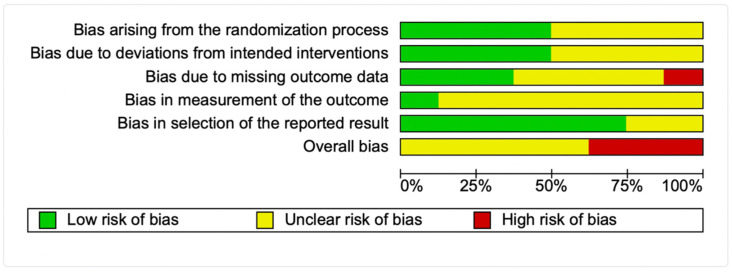
Summary of the risk of bias assessment according to the Cochrane risk-of-bias tool for randomized trials (RoB-2).

The characteristics of the selected six randomized clinical trials with parallel group design [17,18,19,20,21,22] and two with crossover design [23,24] included in this systematic review and meta-analysis are presented in Table 2. Briefly, only one study recruited healthy volunteers [20], while other studies recruited obese female adolescents [17], hypertensive and dyslipidaemic individuals [18,23,24], older adults with mild cognitive impairment [19], individuals considered at risk of colorectal cancer [21], or obese females [22]. Two studies reported only on female individuals [17,22]. Five studies used as an intervention Brazil nuts [17,19,20,21,22], two used granulated Brazil nuts [23,24] and one used Brazil nut flour [18]. Six studies were conducted in Brazil [17,18,19,22,23,24], one in Australia [21] and one in New Zealand [20]. Trial duration varied from 1.5 to 6 months (Table 2). None of the included studies reported any adverse effects related to the intervention. When considering the overall risk of bias, five studies showed an unclear risk of bias [17,18,20,21,22], while three were subjected to high risk of bias [19,23,24] (Figure 2 and Supplementary Figure S1).

### 3.1. Effect of Brazil Nut Consumption on Selenium Serum/Plasma Levels

**Figure 3:**
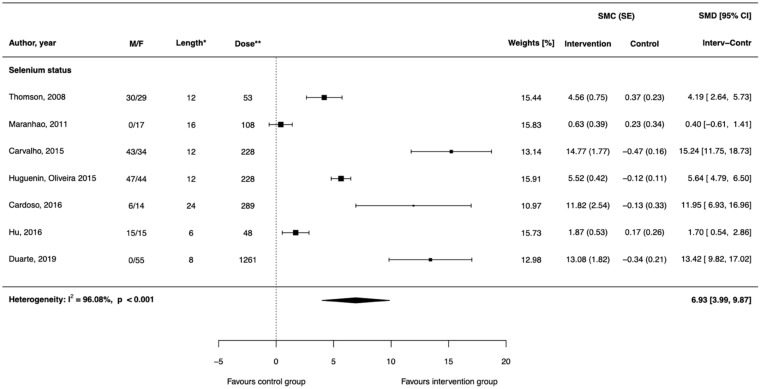
Differences of standardized mean changes in selenium status between intervention groups supplemented with Brazil nuts and control groups in randomized controlled trials. * denotes weeks, ** denotes microgram/day of selenium delivered through Brazil nut intervention. Abbreviations: F (female); M (male); SE (standard error); SMC (standardized mean changes); SMD (difference of standardized mean changes).

Six randomized clinical trials with repeated measures and parallel design [17,18,19,20,21,22] and one with crossover design [23] examined the effect of Brazil nut ingestion on selenium concentration in serum or plasma. A significant positive effect of Brazil nuts on selenium blood concentration (SMD = 6.93, 95% CI: 3.99; 9.87) was found (Figure 3, Table 3). The alternative meta-analysis restricted only to six trials with parallel design showed similar results (SMD = 7.33, 95% CI: 3.64; 11.01; Supplementary Figure S2 and Table S3). The estimated effect size was stable irrespectively of the imputed value of missing correlations (Supplementary Table S2). In all models, high heterogeneity was detected (I2 > 95%), funnel plots showed asymmetry, and Egger’s test indicated the possibility of the existence of small-study effects (Supplementary Figure S3). The influential analysis did not reveal a large impact of excluding the individual studies on heterogeneity or overall effect as shown in Table 4. Meta-regression analysis showed that studies with larger sample size that were conducted on older participants and were more recently published depicted a stronger positive effect of the intervention (Supplementary Table S3).

Regarding the effect of Brazil nut consumption on selenium blood level, the findings across all six included studies with parallel design were comparable [17,18,19,20,21,22]. In all randomized clinical trials, the increase in plasma selenium was statistically significant for the Brazil nut group but not for the placebo group. Moreover, the comparison of the overall change in Brazil nut group differed significantly from the placebo group in all studies except one [17]. In one study [20], additionally to Brazil nuts, the supplementation with selenomethionine was examined, revealing the significant difference between each intervention group and placebo group, but supplemented groups did not differ from each other. In two studies [18,20], the measurement of selenium was performed for many time points (at each two or 4 weeks of follow-up), and both absolute selenium concentration and selenium changes differed significantly between intervention and control group at all follow-up time points. In a crossover clinical trial examining hypertensive and dyslipidaemic patients, it has been observed that granulated Brazil nut intake significantly increased plasma selenium concentration by 119% during the 12-weeks lasting interventions with four-week washout interval periods [23]. However, part of the study group who, in the first phase, received granulated Brazil nuts did not return to the same basal values after washout period, causing plasma selenium initial value to be elevated when compared to the other group, and therefore the occurrence of the carry-over effect after the washout period cannot be definitively ruled out.

### 3.2. Effect of Brazil Nut Consumption on Glutathione Peroxidase Activity

**Figure 4:**
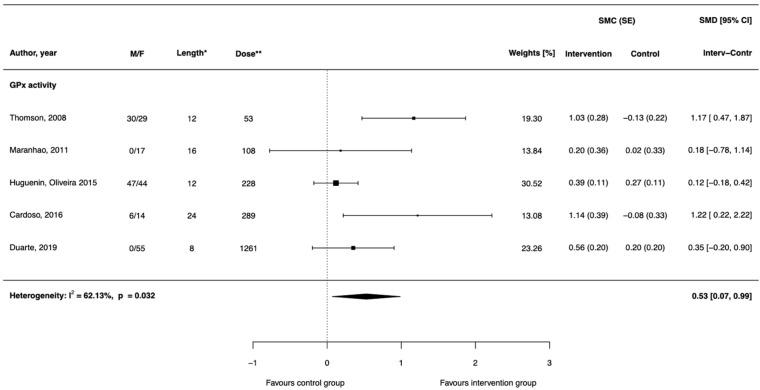
Differences of standardized mean changes in GPx activity between intervention groups supplemented with Brazil nuts and control groups in randomized controlled trials. * denotes weeks, ** denotes microgram/day of selenium delivered through Brazil nut intervention. Abbreviations: F (female); M (male); SE (standard error); SMC (standardized mean changes); SMD (difference of standardized mean changes).

Four randomized clinical trials with parallel design [17,19,20,22] and one with crossover design [23] examined the effect of Brazil nut supplementation on serum, plasma or erythrocyte glutathione peroxidase activity. The effect size assessed based on both dependent- and independent-group trials revealed a positive effect of Brazil nut consumption on glutathione peroxidase (SMD = 0.53, 95% CI: 0.07; 0.99; Figure 4, Table 3). Excluding one crossover trial [23] strengthened the results (SMD = 0.70, 95% CI: 0.19; 1.22; Supplementary Figure S4 and Table S3). Pooled SMD did not substantially change for alternatively imputed values of missing correlations (Supplementary Table S2). Influential analysis showed that the exclusion of three studies [19,20,22] weakened the strength of the effect of Brazil nuts on GPx such that the result was marginally significant (Table 4). Funnel plots together with Eager’s test did not indicate an existence of publication bias (Supplementary Figure S3). Meta-regression analysis showed that none of the potential moderators influenced the association between Brazil nut consumption and GPx activity (Supplementary Table S3).

Among the four eligible parallel-design clinical trials, three reported that the increase in GPx during follow-up was significant for the Brazil nut group, whereas in the placebo group, nonsignificant change was observed (increase or decrease) [19,20,22]. In one study, GPx did not change during the follow-up in either group [17]. Regarding clinical trials with dependent groups design, the result of one identified study showed significantly increased GPx activity after Brazil nut intake [23].

### 3.3. Effect of Brazil Nut Consumption on Serum Lipids Profile

Three studies [17,18,24] examined alteration of serum lipid profile as a consequence of Brazil nut intervention. All pooled results were nonsignificant, including results for total cholesterol (SMD = −0.22, 95% CI: −0.57; 0.14; Table 5, Supplementary Figure S5), HDL cholesterol (SMD = −0.04, 95% CI: −0.28; 0.19; Table 5, Supplementary Figure S6) and LDL cholesterol (SMD = −0.15; 95% CI: −0.43; 0.13, Table 5, Supplementary Figure S7). Alternative imputed missing correlations did not show any significant differences (Supplementary Table S4).

When considering the results of individual studies, in one trial [17], there were no significant inter-group differences at the start of the trial in any of laboratorial variables, but at the end of follow-up, lower values of total and LDL cholesterol as well as TG for the intervention group supplemented with Brazil nuts compared to the placebo group were observed. However, in studies examining hypertensive and dyslipidaemic patients, no significant differences in serum lipids between Brazil nut and control group were detected [18,24].

### 3.4. Effect of Brazil Nut Consumption on Other Markers of Oxidative Stress and Plasma Antioxidant Activity

Three randomized clinical trials investigated the impact of Brazil nut supplementation on markers of oxidative stress, two with parallel design [17,19] and one with crossover design [23]. A study conducted on obese female adolescents [17] reported significantly lower serum levels of oxidized LDL (LDL-ox) in the Brazil nut group compared to the placebo group at the end of the follow-up period. However, no significant changes were observed for 8-epi PGF2α [17]. Another study [23] showed a reduction in LDL-ox by 3.2% after 3 months of Brazil nut intervention in hypertensive and dyslipidaemic patients as well as a significantly lower plasma total antioxidant capacity (TAC) in the placebo group compared to Brazil nut group. Similar to the previous study, there were no statistically significant changes in 8-epi PGF2α. In a study conducted on adults with mild cognitive impairment, no significant changes in MDA, a naturally occurring genotoxic product of lipid peroxidation, after 6 months of daily supplementation with Brazil nuts were observed [19].

### 3.5. Effect of Brazil Nut Consumption on Markers of Inflammation

Among the markers of inflammation that potentially might be affected by Brazil nut consumption, CRP was investigated in three trials [17,21,22]. However, no effect of intervention with Brazil nuts on CRP levels was observed [17,21,22].

## 4. Discussion

In this study, the impact of Brazil nut consumption on selenium status, oxidative stress biomarkers and blood lipids has been evaluated through comprehensive pooled analysis of results from existing randomized clinical trials. The findings of this study provide certain evidence of the effects of Brazil nuts in influencing selenium status and improving antioxidant defences through impact of glutathione peroxidase but no significant effects on blood lipid profile or any other outcome investigated.

Over the last decade, tree nut consumption has been studied in relation to mortality and risk of various non-communicable diseases, including type 2 diabetes, metabolic syndrome, and cardiovascular disease [12]. There are several hypotheses supporting the rationale for various mechanisms of protection, including the content in fibre, minerals, vitamins and antioxidants [25,26,27]. However, the diversity of various tree nuts characterize with peculiar features each type of fruit: among them, Brazil nuts are the only nut providing a considerable amount of selenium. On average, in included studies, supplementation with Brazil nuts per day varied between 5 and 20 g and provided in all except one study selenium intake in the range of about 50–300 microgram/day, increasing the selenium levels to more than the recommended dose (about 50 microgram/day) but within the upper tolerable level (400 microgram/day).

From a mechanistic point of view, selenium is an essential element for cellular functions which is ingested from food sources, absorbed in the gut, carried to the liver to be metabolized, and then transported and distributed to the body’s tissues [28]. Selenium is incorporated into important amino acid derivatives, including selenomethionine, selenocysteine, methylselenocysteine, and selenocystathionine, which play vital roles in the enzymatic systems in which they participate, amongst others, in the catalysis of various oxidation–reduction reactions [29]. Adequate levels of dietary selenium and its efficient incorporation into selenoproteins are essential in the maintenance of the homeostasis [29,30] Selenium modulates body functions through various pathways, such as regulation of immunity functions, reduction of oxidative stress, and prevention of DNA damage [31]. The evidence suggests that selenium can modulate the pathology that accompanies chronic inflammation-related diseases [31,32]. Selenium deficiency and suppressed selenoprotein expression have been implicated in higher levels of inflammatory cytokines in a variety of tissues [29]. Insufficient dietary supplies of selenium can also be responsible for abnormal calcium deposition, heart and skeletal muscle myopathy, and increased risk of developing atherosclerotic plaque within the arteries [33]. Furthermore, it seems that selenium has an antihypertensive effect [34].

The results of the present meta-analysis revealed that Brazil nut supplementation may not only improve selenium status but may also affect glutathione peroxidase activity. Selenium in the form of selenocysteine is present in the active centre of GPx, which represents one of the most important antioxidant enzyme systems with superoxide dismutase and catalase [35]. This activity is due to the presence of a rare amino-acid residue selenocysteine in the catalytic site [36]. Besides acting against accumulation of reactive oxygen species and cell death [37], GPx also influences inflammatory processes linked to oxidative reactions, such as inhibition of NF-kB activation, regulation of prostaglandins and leukotrienes production, and the mitochondrial death pathway [35]. Together, with its potential role in the aforementioned conditions (i.e., CVD and cancer), GPx has also been studied as a neuromodulator in neuropsychiatric and neurodegenerative conditions: stress, bipolar disorder, schizophrenia drug intoxication, Parkinson’s disease, Alzheimer’s disease and others [38].

Although the evidence from experimental studies suggests implication of selenium and glutathione peroxidase in the prevention of lipid peroxidation [39], no effect of Brazil nut intervention on lipid peroxidation marker (8-iso-prostaglandin F2α) was found [17,23]. Similarly, no significant results from this meta-analysis were found when considering blood lipid levels, including results for total cholesterol, HDL cholesterol and LDL cholesterol.

The results of the present meta-analysis should be considered in light of some limitations. First, in crossover designed studies, part of the study participants receiving Brazil nut supplementation in the first step may not have returned to the same basal values, causing initial selenium values to be elevated; therefore, the occurrence of the carry-over effect after the washout period cannot be definitively ruled out. Second, data on correlations between measurements before and after the intervention were not available in all studies, although we tested robustness of the findings, imputing a wide range of possible values of correlations (0.3, 0.5, 0.7) and demonstrating the stability of the results regardless of imputed value. In the analysis of influence of Brazil nut ingestion on selenium concentration, we detected evidence of small-study effects that may be due to not only the publication bias but also other factors such as study quality, differences in test procedures, or patient characteristics.

## 5. Conclusions

In conclusion, the findings from this meta-analysis of RCTs demonstrate that Brazil nut consumption increases selenium blood levels and GPx activity, suggesting their antioxidant effects. However, there is no summary consistent evidence from the scientific literature that this antioxidant effect may translate into improvement of blood lipid profile. This study highlights the need for further research exploring the effect of Brazil nut consumption toward cardiovascular health, taking into account more valid biomarkers.

## Figures and Tables

**Table 1:** PICOS criteria: determination of the population (P), intervention (I), comparison (C), outcomes (O), and study design (S).

PICOS	Description
P (Population)	Men and/or women, adolescents and adults.
I (Intervention)	Brazil nut supplementation (including derivatives, characterized by a similar nutrient profile).
C (Comparison)	Brazil nut supplementation group (alone or combined with other intervention) versus placebo/control group.
O (Outcomes)	Changes in selenium blood levels, oxidative stress and inflammatory markers, and in blood lipid profile.
S (Study design)	Systematic review with meta-analysis.

**Table 2:** Main characteristics of the included randomized clinical trials.

Author, Year	Country	Type and Duration of Intervention, No. of Individuals in Intervention/Control Group	Population	Sex; Mean Age * of Individuals	Measured Outcomes of Interest
**Parallel Design**					
Thomson, 2008 [20]	New Zealand	Brazil nuts (n = 20) vs. placebo (n = 20); 2 nuts/d for 3 months	Healthy volunteers	30M, 29F; 18–60 y	Selenium status, GPx activity
Maranhao, 2011 [17]	Brazil	Brazil nuts (n = 8) vs. lactose (n = 9); 3–5 nuts/d for 4 months	Obese female adolescents	17F; 15.4 ± 2.0 y	Selenium status, GPx activity, cholesterol, HDL-c, LDL-c
Carvalho, 2015 [18]	Brazil	Brazil nut flour (n = 35) vs. placebo flour (n = 42); 13 g/d of nut flour for 3 months	Hypertensive and dyslipidaemic individuals	43M, 34F; 40–80 y	Selenium status, cholesterol, HDL-c, LDL-c
Cardoso, 2016 [19]	Brazil	Brazil nuts (n = 11) vs. normal diet (n = 9); 1 nut/d for 6 months	Older adults with mild cognitive impairment	6M, 14F; 77.7 ± 5.3 y	Selenium status, GPx activity
Hu, 2016 [21]	Australia	Brazil nuts (n = 9) vs. GTE (n=10); 6 nuts/d for 1.5 month	Individuals considered at risk of colorectal cancer	15M, 15F; 52–75 y	Selenium status
Duarte, 2019 [22]	Brazil	Brazil nuts (n = 29) vs. no supplementation (n = 26); 1 nut/d for 2 months	Obese female adults	0M, 55F; 18–55 y	Selenium status, GPx activity
**Crossover Design**					
Huguenin, Oliveira 2015 [23]	Brazil	Diet and placebo (n = 91) vs. diet and GBN (n = 91); 13 g/d GBN for 3 months	Hypertensive and dyslipidaemic individuals	47M, 44F; 62.1 ± 9.3 y	Selenium status, GPx activity
Huguenin, Moreira 2015 [24]	Brazil	Diet and placebo (n = 91) vs. diet and GBN (n = 91); 13 g/d GBN for 3 months	Hypertensive and dyslipidaemic individuals	47M, 44F; 62.1 ± 9.3 y	Cholesterol, HDL-c, LDL-c

Abbreviations: d (day); F (female); GBN (granulated Brazil nuts); GPx (glutathione peroxidase); GTE (green tea extracts); M (male); MCI (mild cognitive impairment); SD (standard deviation); y (years). * Age is reported as mean ± SD, in case data were not available, age range is provided.

**Table 3:** Effect of Brazil nut intervention compared to placebo on selenium status and GPx activity.

Outcome	Number of Studies	SMD (95% CI)	I2 (%)	p_heterogeneity_	τ^2^
**Parallel and crossover design**					
Selenium status	7	6.93 (3.99; 9.87)	96.1	<0.001	11.27
GPx activity	5	0.53 (0.07; 0.99)	62.1	0.032	0.19
**Parallel design**					
Selenium status	6	7.33 (3.64; 11.01)	95.9	<0.001	19.06
GPx activity	4	0.70 (0.19; 1.22)	44.2	0.146	0.12

Abbreviations: CI (confidence interval); GPx (glutathione peroxidase); I2 (the percentage of variation across studies that is due to heterogeneity); SMD (difference of standardized mean changes); τ2 (the absolute value of the heterogeneity).

**Table 4:** Results of influential analysis by excluding one study at a time (results presented for imputed value of missing correlation equal to 0.5).

	Selenium Status		GPx Activity	
Author, Year	SMD (95% CI)	I2 (%)	SMD (95% CI)	I2 (%)
Duarte, 2019	5.88 (2.96; 8.8)	96.0	0.62 (−0.03; 1.26)	71.6
Hu, 2016	8.01 (4.44; 11.59)	96.4	-	-
Cardoso, 2016	6.29 (3.25; 9.34)	96.5	0.42 (−0.04; 0.87)	59.6
Huguenin, Oliveira 2015	7.33 (3.64; 11.01)	95.9	0.70 (0.19; 1.22)	44.2
Carvalho, 2015	5.54 (2.77; 8.3)	95.5	-	-
Maranhao, 2011	8.14 (5; 11.28)	94.8	0.60 (0.06; 1.15)	71.3
Thomson, 2008	7.53 (4.03; 11.03)	96.7	0.31 (−0.05; 0.68)	32.9

Abbreviations: CI (confidence interval); GPx (glutathione peroxidase); I2 (the percentage of variation across studies that is due to heterogeneity); SMD (difference of standardized mean changes).

**Table 5:** Effect of Brazil nut intervention compared to placebo on lipid profile.

Outcome	Number of Studies	SMD (95% CI)	I2 (%)	p_heterogeneity_	τ^2^
**Parallel and Crossover Design**					
Cholesterol	3	−0.22 (−0.57; 0.14)	38.6	0.196	0.04
HDL-c	3	−0.04 (−0.28; 0.19)	00.0	0.872	0.00
LDL-c	3	−0.15 (−0.43; 0.13)	16.2	0.303	0.01

Abbreviations: CI (confidence interval); HDL-c (high-density lipoprotein cholesterol); I2 (the percentage of variation across studies that is due to heterogeneity); LDL-c (low-density lipoprotein cholesterol); SMD (difference of standardized mean changes); τ2 (the absolute value of the heterogeneity).

## Supplementary Materials

The following supporting information can be downloaded at: https://www.mdpi.com/article/10.3390/antiox11020403/s1, Figure S1: Risk of bias assessment of the included studies according to the Cochrane risk-of-bias tool for randomized trials (RoB-2); Figure S2: Differences of standardized mean changes in selenium status between intervention groups supplemented with Brazil nuts and control groups in parallel randomized controlled trials; Figure S3: Funnel plots for meta-analyses of differences of standardized mean changes for selenium status, GPx activity, cholesterol, HDL-c and LDL-c level; Figure S4: Differences of standardized mean changes in GPx activity between intervention groups supplemented with Brazil nuts and control groups in parallel randomized controlled trials; Figure S5: Differences of standardized mean changes in cholesterol level between intervention groups supplemented with Brazil nuts and control groups in randomized controlled trials; Figure S6: Differences of standardized mean changes in HDL-c level between intervention groups supplemented with Brazil nuts and control groups in randomized controlled trials; Figure S7: Differences of standardized mean changes in LDL-c level between intervention groups supplemented with Brazil nuts and control groups in randomized controlled trials; Table S1: Preferred Reporting Items for Systematic Reviews and Meta-Analysis (PRISMA) checklist; Table S2: Effect of Brazil nut intervention compared to placebo on selenium status and GPx activity after imputed alternative values of missing correlations; Table S3: Results of meta-regression analysis (results presented for the imputed value of missing correlation equal to 0.5); Table S4: Effect of Brazil nut intervention compared to placebo on lipid profile after imputed alternative values of missing correlations.
