# Supplementary material for: Effect of Brazil Nuts on Selenium Status, Blood Lipids, and Biomarkers of Oxidative Stress and Inflammation: A Systematic Review and Meta-Analysis of Randomized Clinical Trials
Source: Antioxidants (Basel). 2022 Feb 16;11(2):403. doi: 10.3390/antiox11020403 (PMC8869304; doi:10.3390/antiox11020403)

Supplementary Table S1. Preferred Reporting Items for Systematic Reviews and Meta-Analysis (PRISMA) checklist.

**Section/topic**	**#**	**Checklist item**	**Reported on page #**
**TITLE**			
Title	1	Identify the report as a systematic review, meta-analysis, or both.	1
**ABSTRACT**			
Structured summary	2	Provide a structured summary including, as applicable: background; objectives; data sources; study eligibility criteria, participants, and interventions; study appraisal and synthesis methods; results; limitations; conclusions and implications of key findings; systematic review registration number.	1
**INTRODUCTION**			
Rationale	3	Describe the rationale for the review in the context of what is already known.	1,2
Objectives	4	Provide an explicit statement of questions being addressed with reference to participants, interventions, comparisons, outcomes, and study design (PICOS).	2
**METHODS**			
Protocol and registration	5	Indicate if a review protocol exists, if and where it can be accessed (e.g., Web address), and, if available, provide registration information including registration number.	NA
Eligibility criteria	6	Specify study characteristics (e.g., PICOS, length of follow-up) and report characteristics (e.g., years considered, language, publication status) used as criteria for eligibility, giving rationale.	2,3
Information sources	7	Describe all information sources (e.g., databases with dates of coverage, contact with study authors to identify additional studies) in the search and date last searched.	2
Search	8	Present full electronic search strategy for at least one database, including any limits used, such that it could be repeated.	2,3
Study selection	9	State the process for selecting studies (i.e., screening, eligibility, included in systematic review, and, if applicable, included in the meta-analysis).	2,3 and Figure 1

Data collection process	10	Describe method of data extraction from reports (e.g., piloted forms, independently, in duplicate) and any processes for obtaining and confirming data from investigators.	3
Data items	11	List and define all variables for which data were sought (e.g., PICOS, funding sources) and any assumptions and simplifications made.	2 and Table 1
Risk of bias in individual studies	12	Describe methods used for assessing risk of bias of individual studies (including specification of whether this was done at the study or outcome level), and how this information is to be used in any data synthesis.	3,4
Summary measures	13	State the principal summary measures (e.g., risk ratio, difference in means).	3,4
Synthesis of results	14	Describe the methods of handling data and combining results of studies, if done, including measures of consistency (e.g., I^2) for each meta-analysis.	3,4
Risk of bias across studies	15	Specify any assessment of risk of bias that may affect the cumulative evidence (e.g., publication bias, selective reporting within studies).	3,4
Additional analyses	16	Describe methods of additional analyses (e.g., sensitivity or subgroup analyses, meta-regression), if done, indicating which were pre-specified.	3,4
**RESULTS**			
Study selection	17	Give numbers of studies screened, assessed for eligibility, and included in the review, with reasons for exclusions at each stage, ideally with a flow diagram.	4
Study characteristics	18	For each study, present characteristics for which data were extracted (e.g., study size, PICOS, follow-up period) and provide the citations.	4
Risk of bias within studies	19	Present data on risk of bias of each study and, if available, any outcome level assessment (see item 12).	Figure 2 and Supplementary Figure 1
Results of individual studies	20	For all outcomes considered (benefits or harms), present, for each study: (a) simple summary data for each intervention group (b) effect estimates and confidence intervals, ideally with a forest plot.	Figure 3 and Figure 4
Synthesis of results	21	Present results of each meta-analysis done, including confidence intervals and measures of consistency.	Figure 3 and Figure 4

Risk of bias across studies	22	Present results of any assessment of risk of bias across studies (see Item 15).	Figure 2 and Supplementary Figure 1
Additional analysis	23	Give results of additional analyses, if done (e.g., sensitivity or subgroup analyses, meta-regression [see Item 16]).	Supplementary material
**DISCUSSION**			
Summary of evidence	24	Summarize the main findings including the strength of evidence for each main outcome; consider their relevance to key groups (e.g., healthcare providers, users, and policy makers).	4,5
Limitations	25	Discuss limitations at study and outcome level (e.g., risk of bias), and at review-level (e.g., incomplete retrieval of identified research, reporting bias).	5
Conclusions	26	Provide a general interpretation of the results in the context of other evidence, and implications for future research.	5
**FUNDING**			
Funding	27	Describe sources of funding for the systematic review and other support (e.g., supply of data); role of funders for the systematic review.	6

Supplementary Table S2. Effect of Brazil nut intervention compared to placebo on selenium status and GPx activity after imputed alternative values of missing correlations.

Outcome	Number of studies	Imputed missing correlation = 0.3		Imputed missing correlation = 0.7	
		**SMD (95% CI)**	**I2 (%)**	**SMD (95% CI)**	**I2 (%)**
**Parallel and crossover design**					
Selenium status	7	6.94 (3.99; 9.89)	95.7	6.93 (3.99; 9.86)	96.6
GPx activity	5	0.50 (0.05; 0.96)	49.8	0.55 (0.10; 0.99)	74.5
**Parallel design**					
Selenium status	6	7.35 (3.58; 11.13)	95.7	7.30 (3.71; 10.88)	96.2
GPx activity	4	0.70 (0.19; 1.21)	25.2	0.70 (0.19; 1.22)	63.3

Abbreviations: CI (confidence interval); GPx (glutathione peroxidase); I2 (the percentage of variation across studies that is due to heterogeneity); SMD (difference of standardized mean changes).

Supplementary Table S3. Results of meta-regression analysis (results presented for imputed value of missing correlation equal to 0.5).

	Selenium status					GPx activity				
**Moderator**	**Number of studies**	**Slope**	**$p_{moderator}$**	**I2 (%)**	**$p_{heterogeneity}$**	**Number of studies**	**Slope**	**$p_{moderator}$**	**I2 (%)**	**$p_{heterogeneity}$**
**Parallel and crossover design**										
Year of publication	7	0.88 (-0.01; 1.78)	0.052	96.4	<0.001	5	-0.05 (-0.16; 0.07)	0.439	75.9	0.006
Mean age of participants	7	0.12 (-0.04; 0.28)	0.157	95.4	<0.001	5	0.01 (-0.02; 0.04)	0.475	80.8	0.001
Follow up (weeks)	7	0.16 (-0.5; 0.82)	0.632	97.0	<0.001	5	0.04 (-0.05; 0.14)	0.335	77.6	0.004
Percentage of men	7	0.01 (-0.13; 0.14)	0.916	95.9	<0.001	5	0.01 (-0.02; 0.03)	0.596	80.7	0.001
Mean BMI of participants	5	-0.09 (-1.29; 1.1)	0.878	96.6	<0.001	4	-0.06 (-0.21; 0.09)	0.411	81.3	0.005
Sample size	7	0.08 (-0.02; 0.18)	0.121	93.9	<0.001	5	-0.01 (-0.02; 0.01)	0.458	70.8	0.016
Selenium per day (for 100 microg)	7	0.87 (0.24; 1.51)	0.007	95.1	<0.001	5	-0.03 (-0.15; 0.09)	0.665	80.8	0.001

Abbreviations: BMI (body mass index); GPx (glutathione peroxidase); I2 (the percentage of variation across studies that is due to heterogeneity).

Supplementary Table S4. Effect of Brazil nut intervention compared to placebo on lipid profile after imputed alternative values of missing correlations.

		**Imputed missing correlation = 0.3**		**Imputed missing correlation = 0.7**	
**Outcome**	**Number of studies**	**SMD (95% CI)**	**I2 (%)**	**SMD (95% CI)**	**I2 (%)**
**Parallel and crossover design**					
Cholesterol	3	-0.18 (-0.50; 0.14)	19.6	-0.27 (-0.68; 0.13)	57.7
HDL-c	3	-0.05 (-0.32; 0.21)	0.0	-0.03 (-0.23; 0.17)	0.0
LDL-c	3	-0.13 (-0.39; 0.14)	0.0	-0.18 (-0.50; 0.14)	39.4

Abbreviations: CI (confidence interval); HDL-c (high-density lipoprotein cholesterol); I2 (the percentage of variation across studies that is due to heterogeneity); LDL-c (low-density lipoprotein cholesterol); SMD (difference of standardized mean changes).

Supplementary Figure S1. Risk of bias assessment of the included studies according to the Cochrane risk-of-bias tool for randomized trials (RoB-2).

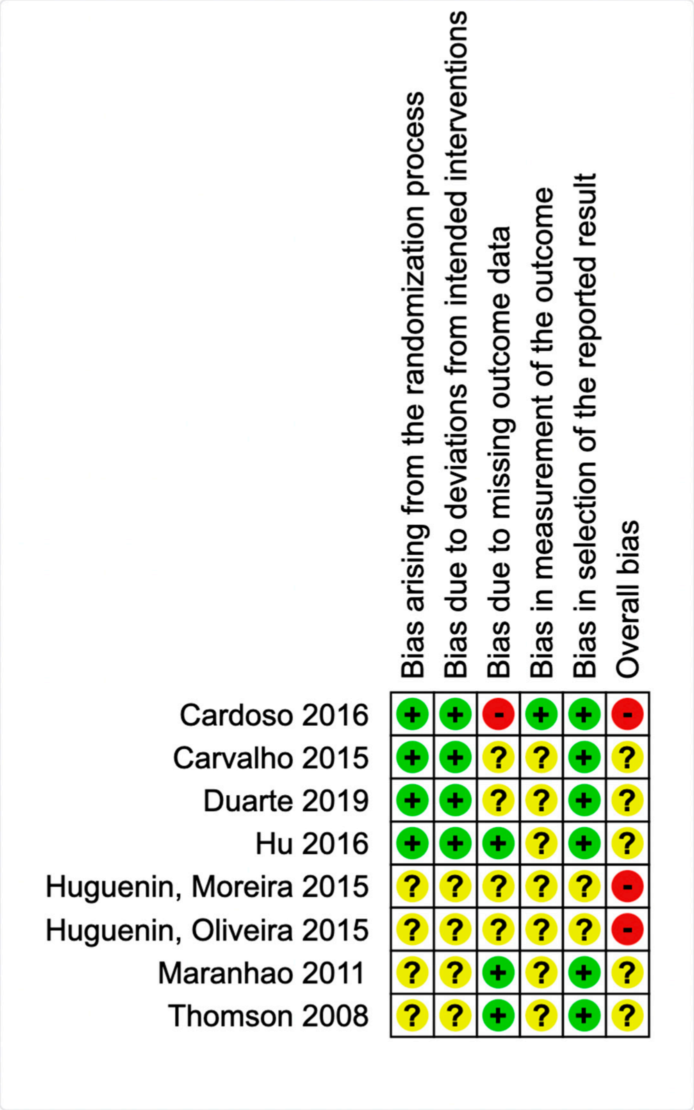

Supplementary Figure S2. Differences of standardized mean changes in selenium status between intervention groups supplemented with Brazil nuts and control groups in parallel randomized controlled trials. * denotes weeks, ** denotes microg/day of selenium delivered through Brazil nut intervention. Abbreviations: F (female); M (male); SE (standard error); SMC (standardized mean changes); SMD (difference of standardized mean changes).

Author, year	M/F	Length*	Dose**	Weights [%]	SMC (SE) Intervention	SMC (SE) Control	SMD [95% CI] Interv-Contr
Selenium status							
Thomson, 2008	30/29	12	53	17.97	4.56 (0.75)	0.37 (0.23)	4.19 [2.64, 5.73]
Maranhao, 2011	0/17	16	108	18.31	0.63 (0.39)	0.23 (0.34)	0.40 [−0.61, 1.41]
Carvalho, 2015	43/34	12	228	15.91	14.77 (1.77)	−0.47 (0.16)	15.24 [11.75, 18.73]
Cardoso, 2016	6/14	24	289	13.81	11.82 (2.54)	−0.13 (0.33)	11.95 [6.93, 16.96]
Hu, 2016	15/15	6	48	18.22	1.87 (0.53)	0.17 (0.26)	1.70 [0.54, 2.86]
Duarte, 2019	0/55	8	1261	15.77	13.08 (1.82)	−0.34 (0.21)	13.42 [9.82, 17.02]
Heterogeneity: I^2 = 95.94%, p < 0.001							7.33 [3.64, 11.01]

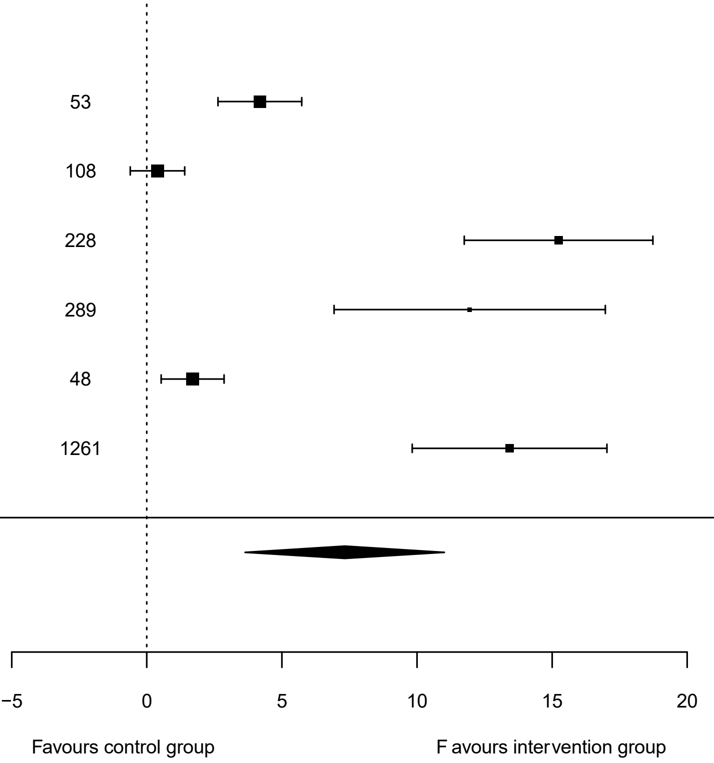

Supplementary Figure S3. Funnel plots for meta-analyses of differences of standardized mean changes for selenium status, GPx activity, cholesterol, HDL-c and LDL-c level.

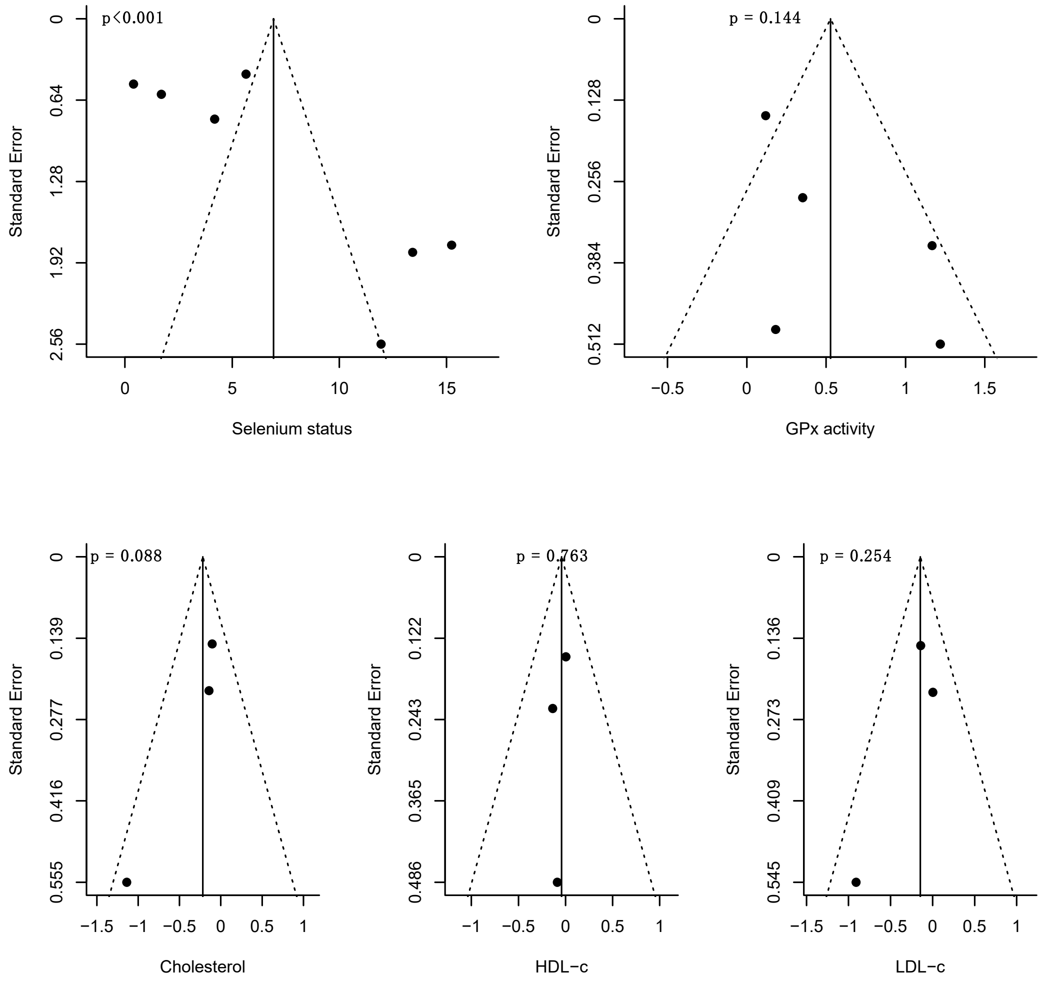

Supplementary Figure S4. Differences of standardized mean changes in GPx activity between intervention groups supplemented with Brazil nuts and control groups in parallel randomized controlled trials. * denotes weeks, ** denotes microg/day of selenium delivered through Brazil nut intervention. Abbreviations: F (female); M (male); SE (standard error); SMC (standardized mean changes); SMD (difference of standardized mean changes).

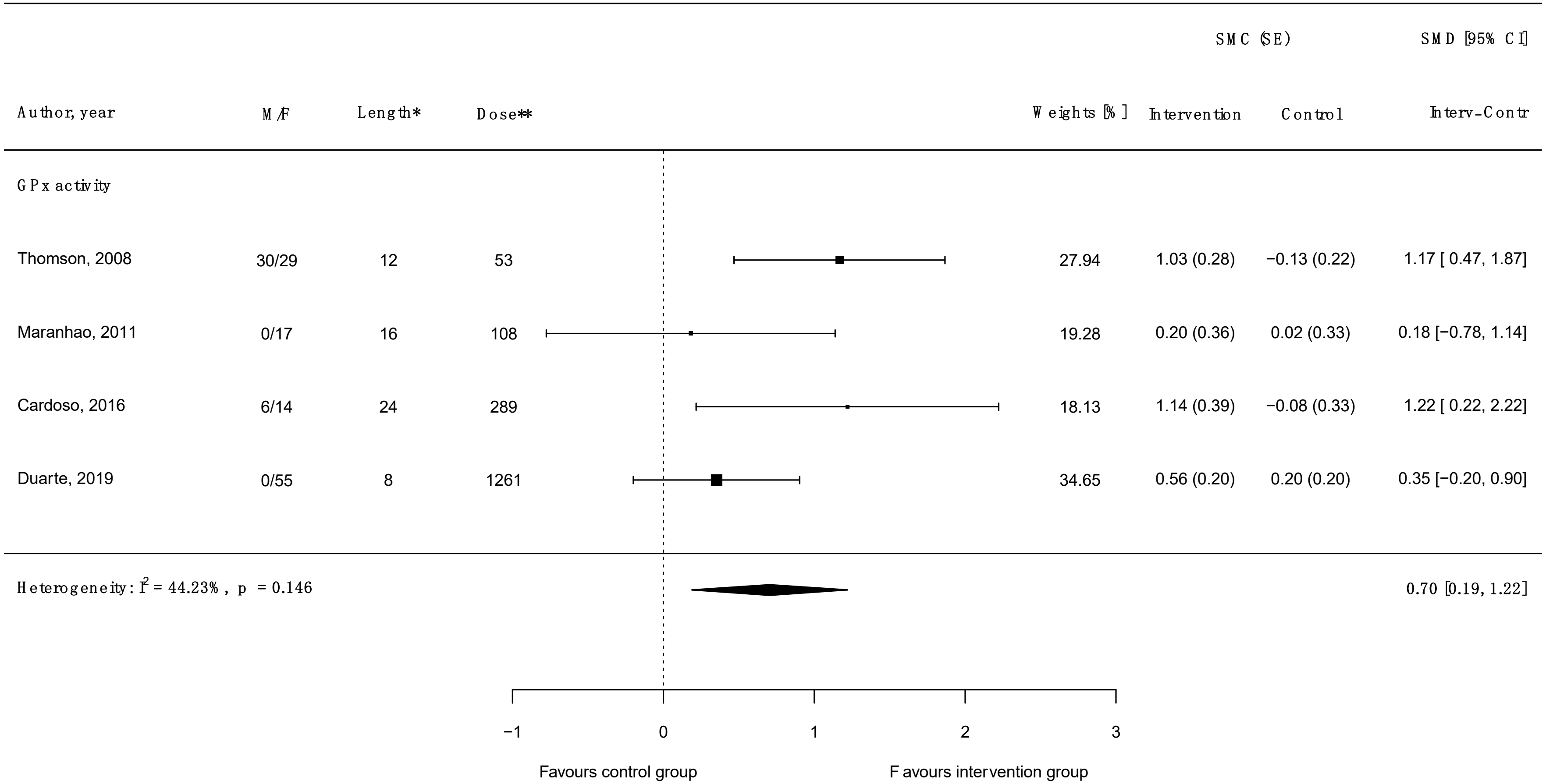

Supplementary Figure S5. Differences of standardized mean changes in cholesterol level between intervention groups supplemented with Brazil nuts and control groups in randomized controlled trials. * denotes weeks, ** denotes microg/day of selenium delivered through Brazil nut intervention. Abbreviations: F (female); M (male); SE (standard error); SMC (standardized mean changes); SMD (difference of standardized mean changes).

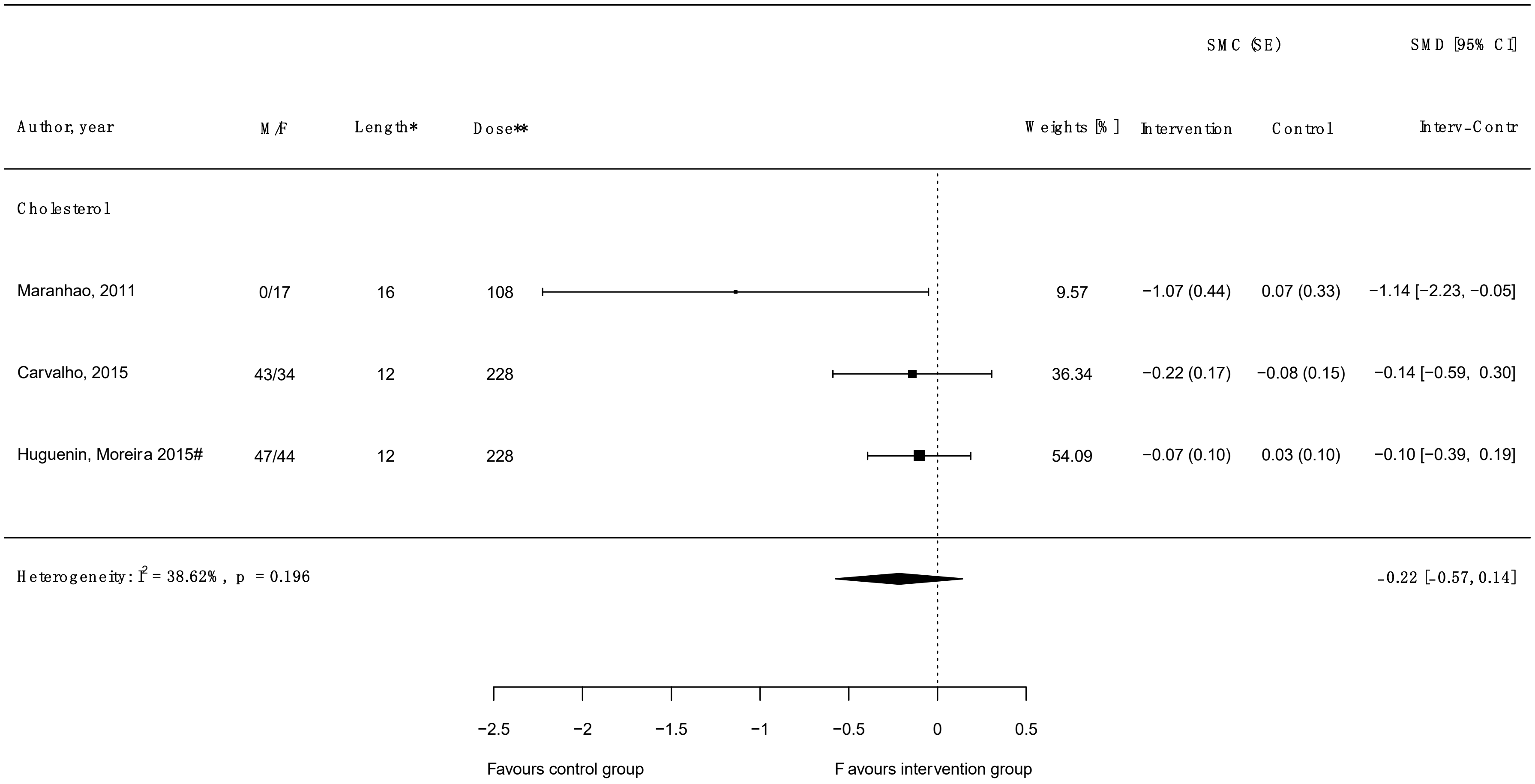

Supplementary Figure S6. Differences of standardized mean changes in HDL-c level between intervention groups supplemented with Brazil nuts and control groups in randomized controlled trials. * denotes weeks, ** denotes microg/day of selenium delivered through Brazil nut intervention. Abbreviations: F (female); M (male); SE (standard error); SMC (standardized mean changes); SMD (difference of standardized mean changes).

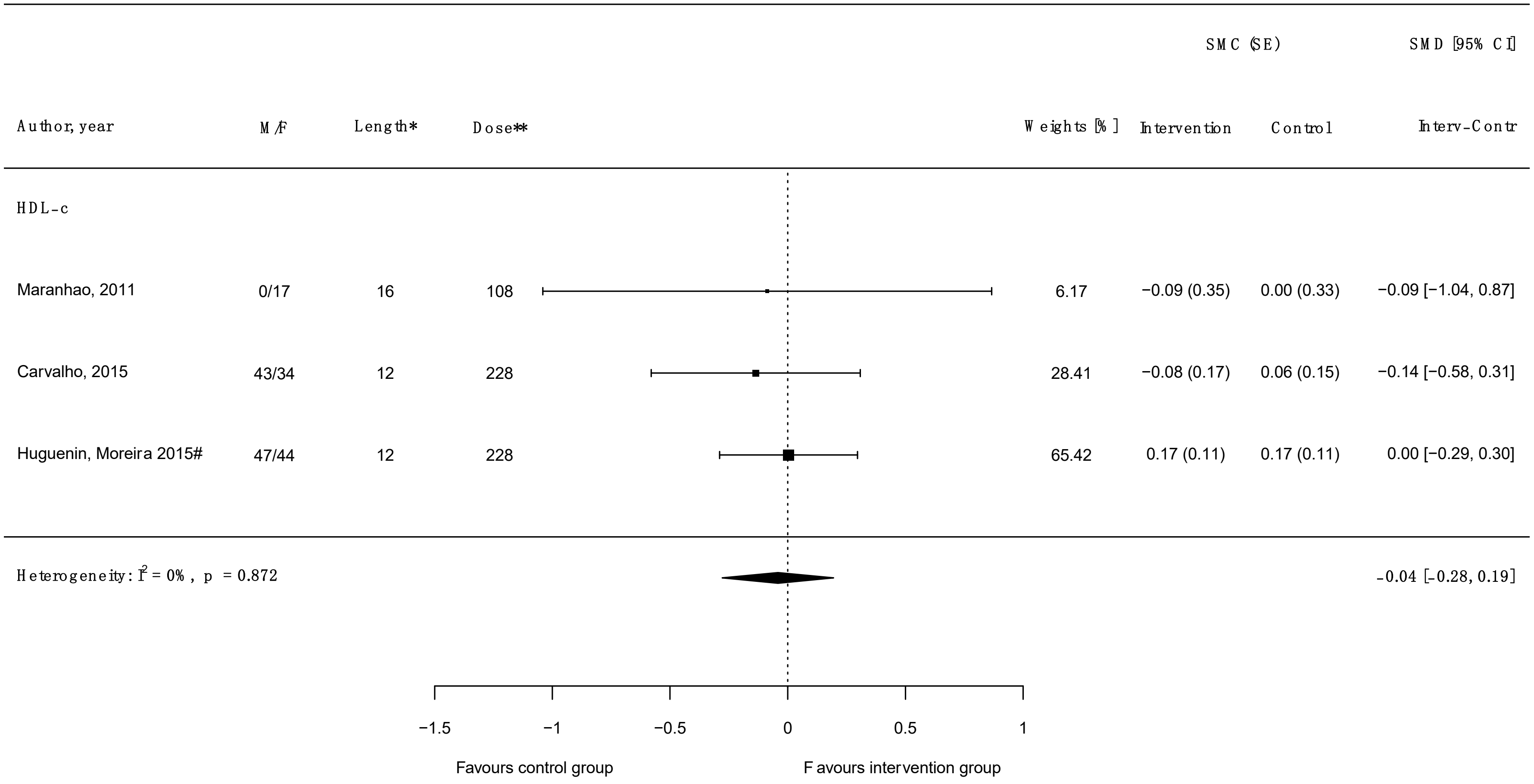

Supplementary Figure S7. Differences of standardized mean changes in LDL-c level between intervention groups supplemented with Brazil nuts and control groups in randomized controlled trials. * denotes weeks, ** denotes microg/day of selenium delivered through Brazil nut intervention. Abbreviations: F (female); M (male); SE (standard error); SMC (standardized mean changes); SMD (difference of standardized mean changes).

Author, year	M/F	Length*	Dose**	Weights [%]	SMC (SE) Intervention	SMC (SE) Control	SMD [95% CI] Interv-Contr
LDL-c							
Maranhao, 2011	0/17	16	108	6.69	−0.99 (0.43)	−0.08 (0.33)	−0.91 [−1.98, 0.16]
Carvalho, 2015	43/34	12	228	32.56	−0.11 (0.17)	−0.11 (0.15)	0.00 [−0.44, 0.45]
Huguenin, Moreira 2015#	47/44	12	228	60.76	−0.12 (0.11)	0.02 (0.10)	−0.14 [−0.43, 0.15]
Heterogeneity: I^2 = 16.18%, p = 0.303							−0.15 [−0.43, 0.13]

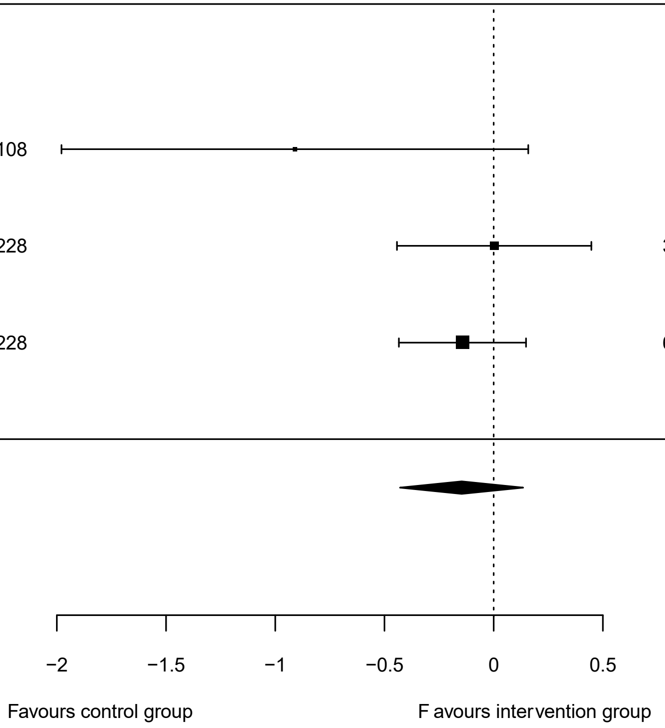

Supplement: Supplementary file 1 [file antioxidants-11-00403-s001.zip › antioxidants-1586812-supplementary.pdf]
